# Sustained prognostic value of proadrenomedulin in severe sepsis and septic shock

**DOI:** 10.1186/2197-425X-3-S1-A792

**Published:** 2015-10-01

**Authors:** R Cicuendez, L Nogales, A Bueno, S Gonzalez De Zarate, D Calvo, C Andres, P Bueno, E Zarca, MF Muñoz, J Bermejo, JM Eiros, F Gandia, D Andaluz-Ojeda

**Affiliations:** Hospital Clínico Universitario Valladolid, Intensive Medicine, Valladolid, Spain

## Introduction

In sepsis, none of the prognostic biomarkers employed have shown the necessary sensitivity and specificity to be used routinely in clinical practice. In this research we analyze the relationship between proADM an other routinely biomarkers employed in the management of severe sepsis with mortality.

## Methods

Prospective observational study. Plasmatic levels of ProADM, C reactive protein (CRP) and procalcitonin (PCT) and lactate in 110 consecutive patients admitted to ICU with severe sepsis / septic shock at days 1^st^, 3^rd^ and 7^th^ after admission. Clinical and demographic data: age, gender, comorbidities, APACHE II and SOFA scores. Recruited period over 12 months. Statistical analysis: χ^2^ test for categorical variables. AUROC for diagnostic prediction of mortality. Spearman Karber test for correlations between severity scores and biomarker levels. Multivariate Cox regression analysis adjusted by age, gender and APACHE II score to assess the impact of variables on mortality across the time. Statistical significance: p < 0.05.

## Results

110 patients. Male: 63%; APACHE II score: 21; SOFA score: 8.5; Septic shock: 86%; ICU mortality: 32.7%; Respiratory focus: 49%; Gram negatives: 33%; Gram positives 30%.

ProADM was the biomarker showing better prognostic accuracy in any time points analyzed by AUROC (p) for 28 day mortality, better than SOFA score at 1^st^ and 7^th^ days of admission: At day 1^st^ proADM = 0.80 (p < 0.001); PCT = 0.62 (p = n.s); lactate = 0.71 (p < 0.001); CRP = 0.47 (p = n.s); SOFA 0.77 (p < 0.001); APACHE II = 0.72 (p < 0.001). At day 3^rd^ proADM = 0.85 (p < 0.001); PCT = 0.68 (p = 0.025); lactate = 0.78 (p < 0.001); CRP = 0.49 (p =n.s); SOFA = 0.86 (p < 0.001). At day 7^th^ ProADM = 0.83 (p < 0.001); PCT = 0.67 (p = n.s); lactate = 0.62 (p = n.s); CRP = 0.61 (p=n.s); SOFA = 0.81 (p = 0.03).

Correlation test showed that proADM present the strongest association with SOFA in all time points analyzed [r; (p)]. At day 1^st^ proADM = 0.71 ( < 0.001); PCT = 0.43 (p < 0.001); lactate = 0.50 (p < 0.001); CRP = 0.52 (p=n.s). At day 3^rd^ proADM = 0.69 (p < 0.001); PCT = 0.51 (p>0.001); lactate = 0.51 (p < 0.001); PCR = 0.23 (p = n.s). At day 7^th^ proADM = 0.63 (p < 0.001); PCT = 0.14 (p=n.s); lactate = 0.40 (p= 0.02); CRP = 0.47 (p=n.s).

Multivariate Cox regression analysis showed proADM as the only biomarker showing association with mortality at day 28^th^ in all time points analyzed [HR; p value; (CI)]. ProADM day 1^st^: [HR=1.085; p = 0.03; CI (1.05-1.170)]; proADM day 3^rd^ [HR=1.051; p = 0.03; CI (1.001-1.104)]; proADM day 7^th^ [HR=1.234; p= 0.001; CI (1.096-1.389)].

## Conclusions

ProADM is a consistent marker of mortality risk and severity along time in severe sepsis and septic shock. This provides a selective advantage over other biomarkers as prognostic tool. The inclusion in clinical practice could help to intensivist, along with the rest of biomarkers and scores of severity, for better optimization in making decisions for management and establish prognosis of this disease.

